# CD127+ CD94+ innate lymphoid cells expressing granulysin and perforin are expanded in patients with Crohn’s disease

**DOI:** 10.1038/s41467-021-26187-x

**Published:** 2021-10-06

**Authors:** L. Krabbendam, B. A. Heesters, C. M. A. Kradolfer, N. J. E. Haverkate, M. A. J. Becker, C. J. Buskens, W. A. Bemelman, J. H. Bernink, H. Spits

**Affiliations:** 1 Amsterdam UMC, University of Amsterdam, Department of Experimental Immunology, Amsterdam Infection & Immunity Institute (AI&II), Cancer Center Amsterdam, Meibergdreef 9, Amsterdam, The Netherlands; 2 Tytgat Institute for Liver and Intestinal Research and Department of Gastroenterology & Hepatology, Amsterdam UMC, University of Amsterdam, Amsterdam, the Netherlands; 3 Amsterdam UMC, University of Amsterdam, Department of Surgery, Amsterdam Gastroenterology & Metabolism (AG&M), Meibergdreef 9, Amsterdam, The Netherlands; 4 Present Address: Department of Pulmonary Medicine, Erasmus MC, Rotterdam, Netherlands; 5 Present Address: Department of Chemical Biology and Drug Discovery, Utrecht Institute for Pharmaceutical Sciences, Utrecht University, Utrecht, Netherlands; 6 Present Address: Hubrecht Institute, Royal Netherlands Academy of Arts and Sciences (KNAW) and UMC Utrecht, 3584 CT Utrecht, The Netherlands

**Keywords:** Chronic inflammation, Innate immune cells, Innate lymphoid cells, Innate immunity

## Abstract

Phenotypic definition of helper ILC1 and NK cells is problematic due to overlapping markers. Recently we showed the identification of cytotoxic ILC3s characterized by expression of CD94. Here we analyse CD127+ ILCs and NK cells in intestinal lamina propria from healthy donors and Crohn’s disease patients and identify two populations of CD127+CD94+ ILCs, designated population A and B, that can be distinguished on the expression of CD117, CD18 and cytotoxic molecules. Population B expresses granulysin, a cytotoxic molecule linked to bacterial lysis and/or chemotaxis of monocytes. Granulysin protein is secreted by population B cells upon stimulation with IL-15. Activation of population B in the presence of TGF-β strongly reduces the expression of cytotoxic effector molecules of population B. Strikingly, samples from individuals that suffer from active Crohn’s disease display enhanced frequencies of granulysin-expressing effector CD127+CD94+ ILCs in comparison to controls. Thus this study identifies group 1 ILC populations which accumulate in inflamed intestinal tissue of Crohn’s disease patients and may play a role in the pathology of the disease.

## Abstract (web summary)

Phenotypic markers that overlap between ILC1 and NK populations have impacted the robust and specific analysis of these immune cell populations. Employing scRNA sequencing here the authors identify CD127^+^ CD94^+^ innate lymphoid cells that express granulysin and perforin and are expanded in patients with Crohn’s disease.

## Introduction

Innate lymphoid cells (ILCs) are enriched at barrier surfaces where they play a pivotal role in the early defense against infiltrating pathogens and contribute to tissue repair and homeostasis^1^. The five canonical subsets of ILCs are cytotoxic NK cells, ILC1s, ILC2s and ILC3s, and Lymphoid-Tissue inducer (LTi) cells. NK cells and ILC1s share their capacity to express the transcription factor T-bet, produce Interferon(IFN)-γ and express many overlapping markers^2–5^. Several groups have described ILC1-like cells displaying cytotoxic capacity similar to NK cells further blurring the distinction between ILC1s and NK cells^2,3^. The use of single-cell RNA sequencing (scRNAseq) and high dimensional flow cytometry profiling has led to the identification of multiple cell types that share phenotypic and functional features with conventional NK cells and helper ILCs in both human^2,6–8^ and mice^9^. Single-cell (sc)RNA sequencing combined with RNA velocity analysis indicated that in the tonsil and intestine ILC3s and intraepithelial CD103+ ILC1s, which express many molecules that are also present in NK cells, represent the ends of a spectrum of intermediate cell populations, reflecting plasticity of these ILC subsets^6^. We and others have identified ILC3 subsets that mediate cellular cytotoxicity^10,11^. These cytotoxic ILC3s are clearly distinct from NK cells and attempts to differentiate the cytotoxic ILC3s into NK cells failed.

In the current study, we analyzed the full spectrum of CD127+ ILCs and CD94+ NK cells in the human intestinal lamina propria from healthy donors and Crohn’s disease patients. Using scRNAseq we identified CD127+CD94+ ILC1s that highly expressed granulysin, a cytotoxic molecule that is linked to bacterial lysis and/or chemotaxis of monocytes^12–16^. These cells were isolated using antibodies against cell surface molecules identified with scRNAseq and further characterization confirmed that these cells highly express granulysin protein, indicating they are in an activated state in vivo. We found that these ILC1s were present in adult but not in fetal intestinal tissue and are dramatically enhanced in frequency in patients that suffer from Crohn’s disease as compared to non-inflamed intestinal tissue.

## Results

### Identification of two populations that co-express CD127 and CD94 in the intestine

**Fig. 1:**
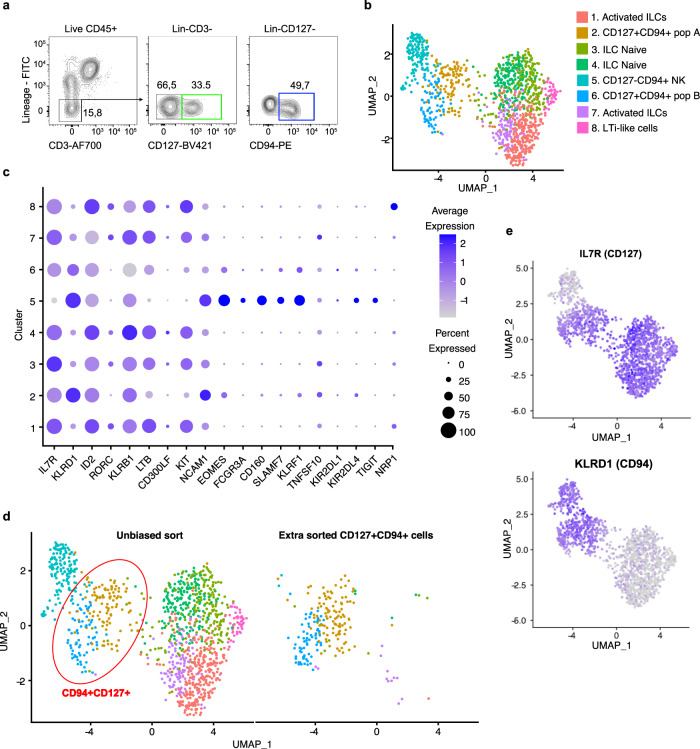
Identification CD127+CD94+ intestinal ILCs. **a** Gating strategy used for sorting of human intestinal ILCs. ILCs and NK cells were gated on viable CD45+ Lineage-(CD1a, CD3, CD4, CD5, CD14, CD19, CD34, CD123, CRTH2, BDCA2, TCRαβ, TCRγδ, and FcER1α) and subsequently on CD127+ for total ILCs and on CD127−CD94+ for NK cells. **b** UMAP clustering of sorted ILCs and NK cells gated for as in (**a**). **c** Expression of indicated genes in clusters identified in (**b**). The color intensity indicates average expression level, and the size of the dot indicated the percentage of cells that express the gene. **d** UMAP clustering of extra sorted CD127+CD94+ cells and clustering of total CD127+ population and CD127−CD94+ population as in (**b**) **e** Expression of IL7R (CD127) and KLRD1 (CD94) plotted in purple on the UMAP clustering. Intensity of purple indicates the expression level.

To explore the full composition of human intestinal ILCs and NK cells, we purified by flow cytometric sorting lineage-CD127+ ILCs (green gate) and CD127−CD94+ NK cells (blue gate) for single-cell RNA sequencing (scRNAseq) from lamina propria mononuclear cells of an inflamed ileal surgical resection specimen (Fig. 1a, full gating strategy in Fig S1, patient characteristics in supplementary table 1). Cells were isolated and sorted directly after surgical removal and 2304 cells were sequenced after which high-quality transcriptomes were obtained from 932 cells and used for further analysis. UMAP clustering revealed 8 clusters (Fig. 1b) of which cluster 5 was *IL7R- KLRD1*+(CD127−CD94+) (Fig. 1c). As expected, these NK cells did not express *RORC* or *KIT*, but expressed *NCAM1, KLRF1, KIRs, FCGR3A, EOMES*, *SLAMF7*, and *CD160* (Fig. 1c).

Cluster 1 and cluster 7 express genes that are associated with ILC3s and ILC2s, such as *IL7R* and low *RORC* levels and are enriched for genes that are involved in transcriptional regulation and cytokine production (*FOS JUNB/D, LIF*), regulation of inflammation (*TNFAIP3*) and wound repair (*AREG*) (Fig. S2a**)**. However, within these clusters no enhanced expression levels of *GATA3* or *IL4R* was detected (Fig. S2b). Like cluster 1 and 7, cluster 3 and 4 expressed helper ILC-related genes (e.g. *IL7R* and *KIT*), but lacked expression of genes that encode for effector functions, and are consequently designated as naïve or non-activated ILCs (Fig. 1c, Fig. S2). Cluster 8 shows similarities to lymphoid-tissue inducer cells (LTi) cells reflected by the expression of *NRP1*^17^ (Fig. 1c) and class II HLA genes (Fig. S2a). *LTB* was clearly expressed in all *IL7R* expressing clusters, but not in the NK cell cluster (Fig. 1c).

Cluster 2 and 6 appeared in between conventional ILC and NK cell clusters and both co-expressed *IL7R* and *KLRD1* and are named CD127+CD94+ population A and population B respectively (Fig. 1b, c). These ILCs resemble cytotoxic ILCs that we recently identified in tonsils^10^. Both CD127+CD94+ population A (cluster 2) and B (cluster 6) weakly expressed NK cell-related genes such as *EOMES, NCAM1* (encoding CD56) and *KIRS*, but not *FCGR3A* (encoding CD16) and shared expression of canonical helper ILCs genes, like *IL7R and KLRB1* (encoding CD161) (Fig. 1c). Gating on CD94+CD127+ cells and subsequent singe cell RNA sequencing of these cells overlaid 87% with cluster 2 and 6 (Fig. 1d) and clearly co-expressed IL7R and KLRD1 (Fig. 1e). Thus, this gating strategy allows to highly purify and capture the fast majority of the CD127+CD94+ ILC populations.

### CD127+CD94+ cells localize in the lamina propria and express NK- and ILC-related genes

**Fig. 2:**
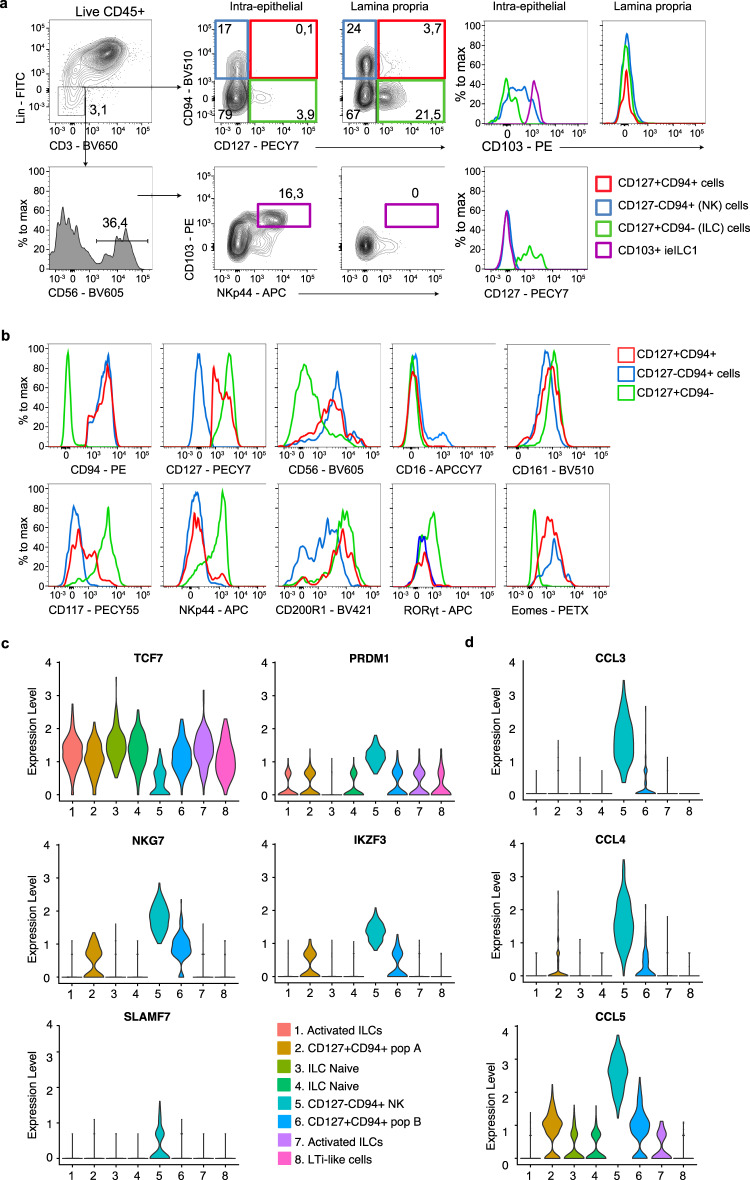
CD127+CD94+ cells localize in the lamina propria and express ILC and NK cell-related markers. **a** Flow cytometry analysis of lymphocytes in the intraepithelial compartment and lamina propria of the intestine. ieILC1 are gated for as live CD45+lineage-CD56+ NKp44+CD103+ (purple gate), NK cells (blue), conventional ILCs (green) and CD127+CD94+ ILCs (red) are gated for as in Fig. 1a. Histograms depict the surface expression of CD103 and CD127 on the indicated populations. **b** Expression of surface phenotypes (CD94, CD127, CD56, CD16, CD161, CD117, NKp44, all at least *N* = 4) and intracellular Eomes (*N* = 3) and RORγt (*N* = 2) of intestinal CD127+CD94+ cells (red), CD127−CD94+ NK cells (blue) and CD127+ CD94− ILCs (green). **c**, **d** Violin plots showing the expression of TCF7, PRDM1, NKG7, IKZF3 (**c**), CCL3, CCL4, and CCL5 (**d**) of clusters identified clusters of Fig. 1.

Examination of their specific anatomical location revealed that CD127+CD94+ ILCs are localized within the lamina propria but do not appear within the intraepithelial compartment and do not express CD103 and are therefore distinct from previously described CD127−CD103+ cytotoxic ILC1s which localize in the epithelium (Fig. 2a)^3,18^. Ex vivo isolated CD127+CD94+ ILCs expressed CD56, CD161, CD200R1 and Eomes, and were partly CD117 positive, but no CD16 and RORγt expression was found, consistent with gene expression patterns (Fig. 2b). Of note, CD200R1 which has been described to distinguish helper ILCs from NK cells in human peripheral blood^19^, and tonsil^10^, as well as in mice^20,21^, was expressed on intestinal CD127−CD94+ NK cells albeit at lower levels than ILCs (Fig. 2b). NK cells expressed more *PRDM1* (encoding Blimp1), *IKZF3* (encoding Aiolos), *NKG7* and *SLAMF7*, whereas *TCF7*, which encodes the transcription factor TCF1, was more abundant in non-NK cells, including CD127+CD94+ ILCs (Fig. 2c). Genes encoding for chemokines CCL3, CCL4, and CCL5 were enriched in NK cells (Fig. 2d). Some expression of activating receptors was observed in population B, but limited to low levels of *NCR1* (encoding NKp46), *KLRF1* and *ITGAL* (encoding CD11a) (Fig. S3a). The inhibitory receptor *CD300A* was expressed in both CD94+CD127+ populations but no expression of other inhibitory receptors such as *KLRG1, CD96*, and *TIGIT* and the tetraspanin *CD81* was found. NK cells did express *TIGIT* (Fig. S3b).

In line with the absence of RORγt, no IL-22 expression was observed in CD127+CD94+ ILCs upon culturing with the IL-22-inducing cytokines IL-23 and IL-1β or IL-15, whereas IFN-γ expression was clearly detected upon IL-15 or IL-12+IL-1β stimulation confirming that CD127+CD94+ cells belong to the group 1 ILCs (Fig. S4).

### Population B highly expresses granulysin and lacks CD117

**Fig. 3:**
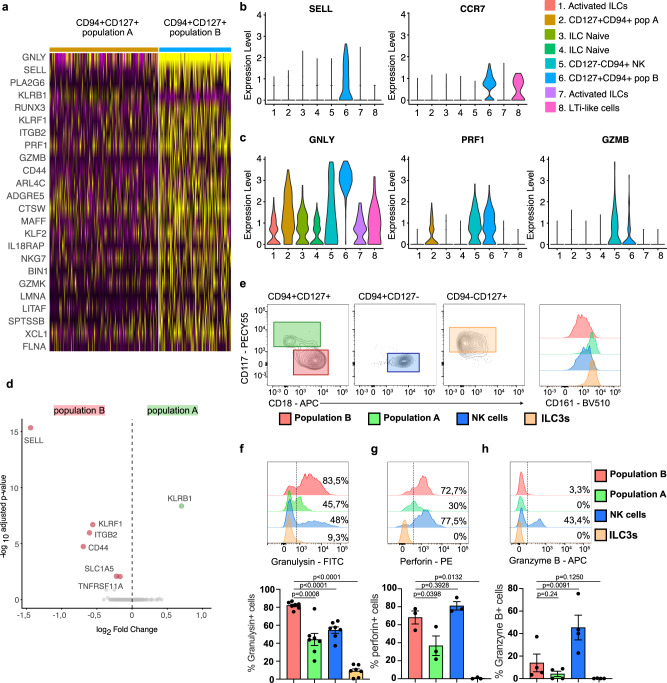
Population B highly expresses granulysin and lacks CD117. **a** Heatmap of significant different expressed genes between cluster 2 (CD127+CD94+ population A) and cluster 6 (CD127+CD94+ population B) **b**, **c** Violin plots showing the expression of SELL and CCR7 (**b**) and GNLY, PRF1 and GZMB (**c**) of all identifies clusters in Fig. 1. **d** Volcano plots of differentially expressed genes that encode for surface markers between population A and B. Red dots on the left indicate genes that are significantly higher expressed in population B (cluster 6) and green dot on the right indicate the gene that is significantly higher expressed in population A (cluster 2). **e** Surface expression of CD18 and CD117 in CD94+CD127+ cells, CD94+CD127− NK cells and CD94−CD127+ ILCs. Colored gated indicate population b (Red), population a (green), NK cells (blue) and ILC3s (orange). The histogram shows the surface expression of CD161 and of these populations. Data is representative of at least *N* = 4. **f**–**h** Flow cytometry analysis of intracellular protein expression of granulysin (*N* = 7), granzyme B (*N* = 4) and perforin (*N* = 3) in indicated populations. Bar graphs show the mean expression ±SEM of the percentage positive cells per population and each dot represents one donor. Data were tested for normal distribution using the Shapiro-Wilk test. All normally distributed data were tested with a paired t-test and not normally distributed data (Granzyme B in ILC3s) was tested with a Wilcoxon matched-pairs rank test. All *p* values are two-sided.

Further dissecting the differences between the observed heterogeneity within the CD127+CD94+ population we first performed differential gene expression (DEG) analysis between population A (cluster 2) and population B (cluster 6) (Fig. 3a). *SELL* (encoding CD62L) and the tissue residency marker *CCR7* are specifically expressed in CD127+CD94+ population B (Fig. 3a, b). Furthermore, this population exclusively and abundantly expressed high levels of *GNLY*, encoding for the cytotoxic protein granulysin (Fig. 3a). *GNLY* expression in population B was much higher than in NK cells and other conventional helper ILC subsets (Fig. 3c). The expression of *PRF1* was the same in CD127+CD94+ population B and NK cells and *GZMB* was lower in population B. CD127+CD94+ population A did not express these cytotoxic molecules (Fig. 3c). Additionally, population B expressed low levels of *GZMK*, but not *GZMA*, *GZMH*, or *GZMM* (Fig. S5).

Next, we asked whether the CD127+CD94+ subpopulations A and B found in clusters 2 and 6 could be separated from each other by flow cytometry using surface markers. DEG analysis between clusters 2 and 6 on surface markers (Fig. 3d) identified several candidates, including SELL (encoding CD62L), CD44, KLRF1 (encoding NKp80), ITGB2 (Encoding CD18) and KLRB1 (encoding CD161). CD62L did not provide consistent expression patterns and CD44 and NKp80 are sensitive for enzymatic digestion^22,23^, and thus cannot be used to separate populations A and B. CD18 was consistently and specifically expressed on part of the CD127+ CD94+ ILCs (Fig. 3e). High expression of CD18 correlated with the absence of CD117 in freshly isolated CD127+CD94+ cells (Fig. 3e). Furthermore, *KLRB1* (encoding for CD161) was more abundant on CD127+CD94+ population A (Fig. 3d) and surface staining confirmed that CD117+CD18dim cells within the CD127+CD94+ population were higher for CD161 compared to CD117-CD18high cells (Fig. 3e). Thus, we identified cluster 6 as CD127+CD94+ CD161dimCD117− population B and cluster 2 as CD127+CD94+ CD161brightCD117+ population A.

The determination of a set of cell surface markers to distinguish the two CD127+CD94+ populations from each other and from NK cells enabled us to confirm the expression of granulysin protein in freshly isolated population B as compared to population A and NK cells of several donors (Fig. 3f). Anti-granulysin stained population B and NK cells with the same mean fluorescent intensity (MFI), and bound substantially lower to population A (Fig. S6a). However, the proportion of granulysin positive cells was much higher in population B than in NK cells (Fig. 3f). In agreement with the scRNAseq results, both NK cells and cells in population B expressed high perforin protein levels but only NK cells expressed granzyme B (Fig. 3g, h). Both in population B and NK cells all granulysin positive cells co-expressed perforin. NK cells contain a larger percentage of perforin+ granulysin− cells (Fig S6b). Of note, CD16− and CD16+ NK cells expressed similar levels of granulysin (Fig. S6c) and CD200R1− and CD200R1+ NK cells did not differ substantially in their mRNA expression of *PRF1*, *GZMB*, and *GNLY (*Fig. S6d).

Thus, CD18 expressing CD127+CD94+CD117- population B is a major innate source of the bactericidal and cytotoxic protein granulysin.

### CD127+CD94+ cells are enriched in inflamed tissue from Crohn’s disease patients

**Fig. 4:**
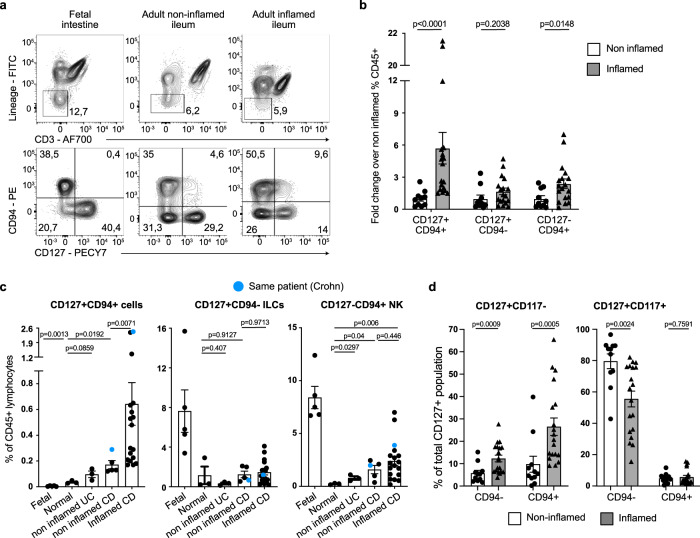
Granulysin-expressing CD117− population B is expanded in inflamed intestine from Crohn’s disease patients. **a** Expression pattern of CD94 and CD127 in live CD45+ lineage-CD3− cells (similar as in Fig. 1a) in fetal intestine, adult macroscopically non-inflamed en inflamed intestine. **b** The fold increase of indicated subsets in inflamed Crohn’s Disease (CD) specimen (*N* = 18) over the average of the frequency of non-inflamed specimen (*N* = 11, containing normal (*N* = 3), macroscopically non-inflamed Ulcerative Colitis (UC) (*N* = 3) and non-inflamed CD intestine (*N* = 5)). Bar graphs show the fold increase ±SEM. Each dot represents one donor. **c** Bar graphs depict indicated populations as a percentage ±SEM of live CD45+ lymphocytes in fetal intestine (*N* = 5), normal intestine (*N* = 3), macroscopically non-inflamed ulcerative colitis (CU) intestine (*N* = 3), non-inflamed Crohn’s Disease (CD) intestine (*N* = 5) and inflamed CD intestine (*N* = 18). Each dot represents one donor, and the blue dots indicate resection samples from the same patients. **d** Bar graphs depict the mean frequency ±SEM of indicated populations as a percentage of the total CD127+ population. The inflamed CD specimen are *N* = 18 and the non-inflamed specimen are *N* = 11 (containing normal (*N* = 3), macroscopically non-inflamed Ulcerative Colitis (UC) (*N* = 3) and non-inflamed CD intestine (*N* = 5). Each dot represents one donor. All data were tested for normal distribution using the Shapiro–Wilk test. All normally distributed data were tested with an unpaired t-test and not normally distributed data were tested with a Mann-Whitney test. All p values are two-sided.

Crohn’s disease (CD) is associated with dysbiosis and enhanced bactericidal and other effector protein secretion of resident and infiltrating immune cells^24,25^. We examined the frequency of conventional CD94−CD127+ ILCs, conventional CD94+ CD127− NK cells and CD94+ CD127+ ILCs in ileal and colonic resection specimen of macroscopically inflamed and non-inflamed tissue from CD or UC patients or from patients with a non-IBD diagnosis (patient information; Supplementary table 1). Although the total pool of CD127+ CD94− conventional helper ILCs did not increase in frequency, we observed an average 6-fold increase of CD127+CD94+ cells and doubling of CD127−CD94+ NK cells within CD45+ Lamina Propria Mononuclear Cells (LPMCs) of non-inflamed vs. inflamed resection specimen of individuals with Crohn’s disease (Fig. 4a, b). The group of non-inflamed controls could be subdivided into samples from patients that did not suffer from any inflammatory disease, but resection was performed due to for example incontinence or radiation damage of which we obtained a non-affected part of the tissue (normal group), three samples were from Ulcerative Colitis (UC) patients who received an ileal pouch, underwent stoma removal or displayed dysplasia, which requires removal of a non-inflamed part of the ileum and four samples were macroscopically non-inflamed from Crohn’s disease (CD) patients as examined by the pathologist. CD127+ CD94+ cells were absent in the fetal intestine and the proportion was very low in the healthy resection specimen and non-inflamed ulcerative colitis (UC) specimen (Fig. 4c). A significant increase in the frequency of both CD127+CD94+ cells and NK cells was observed in inflamed CD intestine when compared to normal non-inflamed specimen (Fig. 4c). However, when comparing non-inflamed samples from CD patients to inflamed samples of CD patients, we did not observe a significant increase in NK cell frequencies, whereas CD127+CD94+ cells were highly increased, suggesting that the inflammation status within the pool of CD patients only affected the frequency of CD127+CD94+ cells and not of NK cells (Fig. 4c). CD117− Population B was responsible for the increase in CD127+CD94+ ILC observed in inflamed tissue vs non-inflamed tissue (Fig. 4d).

The inflammation status of the intestines did not affect the frequency of the total conventional CD127+CD94− ILC pool (Fig. 4b, c), but shifted towards CD127+CD94−CD117− helper ILC1s (Fig. 4d) in line with previous reports^18,26^. Thus, granulysin producing CD127+CD94+CD117−ILCs accumulated in inflamed resection specimen from Crohn’s disease patients, but not at the cost of conventional helper ILCs.

### IL-15 upregulates granulysin in CD127+CD94+ ILC population A and effector functions are blocked by TGF-**β**

**Fig. 5:**
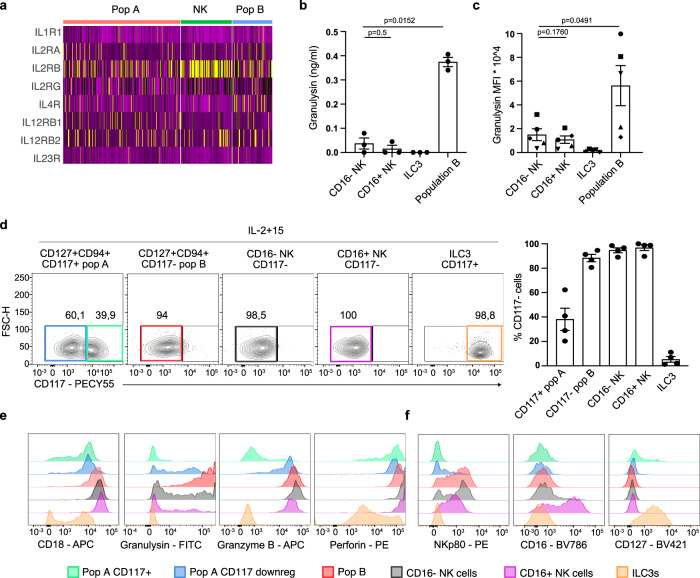
IL-15 induces granulysin secretion in population B and granulysin upregulation in population A. **a** Heatmap of cytokine receptor genes of cluster 2 (CD127+ CD94+ population A), cluster 6 (CD127+CD94+ population B) and cluster 5 (NK cells). **b** Bar graph depicts the mean concentration ±SEM of granulysin in the supernatant of cultured indicated subsets on OP9neo for 7 days in the presence of IL-2 (10U/ml) and IL-15 (50 ng/ml) measured by ELISA (Each dot represents one donor, *N* = 3). **c** Bar graph depicts the mean MFI ± SEM of intracellular granulysin protein of cultured indicated subsets on OP9neo, which were excluded for analysis by gating for live CD45+ cells as depicted in supplemental Fig. 4a, for 7 days in the presence of IL-2 (10U/ml) and IL-15 (50 ng/ml) measured by flow cytometry (Each dot represents 1 donor and matched shapes are the same donor, *N* = 5). **d** Flow cytometry analysis of cultured cells from population A (CD127+ CD94+CD117+), population B (CD127+CD94+CD117−), CD16− NK (CD127−CD94+), CD16+ NK and ILC3s (CD127+CD94−CD117+) for the expression of CD117. Cells were cultured for 7 days on OP9neo, which were excluded from the analysis by gating for live CD45+ cells, in the presence of IL-2 (10U/ml) and IL-15 (50 ng/ml). Dot plot depicts the mean percentage ±SEM of CD117− cells after culturing (each dot represents one donor, *N* = 4). **e**, **f** Representative flow cytometry analysis of surface markers CD16, CD127 (*N* = 4), NKp80 (*N* = 3) and CD18 (*N* = 2 for population A, B, and ILC3 and *N* = 1 for NK cells) and intracellular cytotoxic molecules granulysin (*N* = 5), granzyme B (*N* = 4) and perforin (*N* = 2) of populations gated for as in (**a**). Colors of histograms correspond with the colors of the gates in (**c**). Data were tested for normal distribution using the Shapiro-Wilk test and tested with a paired t-test. All p values are two-sided.

To investigate which cytokines could trigger the CD127+CD94+ populations, we analyzed the expression of cytokine receptors. *IL2RB*, encoding for the beta subunit of the IL-2 and IL-15 receptor, was expressed in both populations A and B, but highest in NK cells (Fig. 5a). Stimulation with IL-15 has been shown to induce granulysin expression and secretion^15^. Indeed, cultures of freshly isolated CD127+CD94+ CD117− ILCs (population B) from LPMCs on OP-9 cells in the presence of low IL-2 and IL-15, released substantial amounts of granulysin protein as compared to NK cells and ILC3s (Fig. 5b) whereas their intracellular protein expression remained high after culture (Fig. 5c).

To further investigate the lineage relationship between the two CD127+CD94+ intestinal ILC populations, we cultured both subsets in the presence of IL-15, which is known to drive a cytotoxicity-inducing program in T and NK cells^27–29^. IL-15 induced CD18 on CD117+ population A in parallel with upregulation of granulysin and downregulation of CD117 (Fig. 5d, e), whereas CD117- population B cells cultured in IL-15 maintained their intracellular granulysin expression. Moreover, IL-15 triggered upregulation of granzyme B and perforin in population A (Fig. 5e). Thus, population A gained functions and phenotypic features of population B under the influence of IL-15 in vitro. In contrast to ILC3s, intestinal CD94+ ILCs downregulated CD127 expression in culture and gained NKp80 expression. ILC3s and CD127+CD94+ ILC populationss, however, did not acquire CD16 (Fig. 5f).

**Fig. 6:**
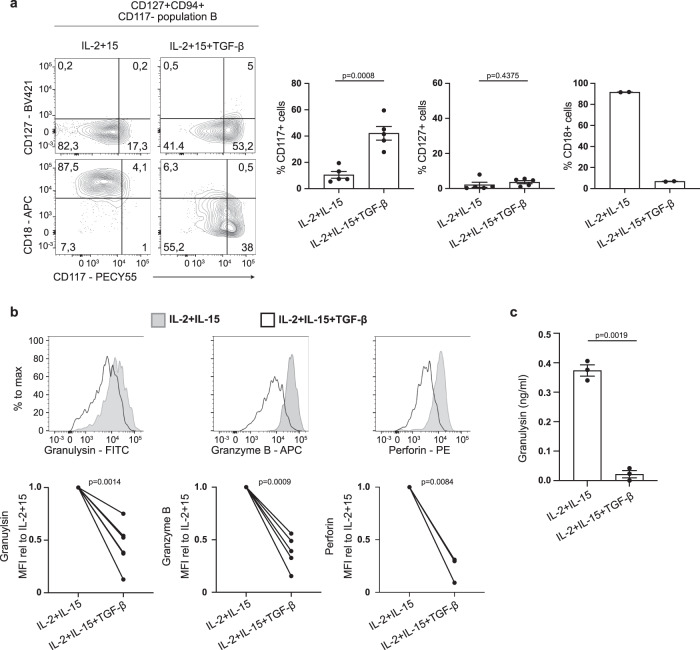
TGF-β impairs granulysin expression and secretion in population B. **a** Flow cytometry analysis of surface expression of CD127, CD117 and CD18 in population B (CD127+CD94+CD117−). Cells were cultured for 7 days on OP9 cells, which were excluded from the analysis by gating for live CD45+ cells as depicted in supplemental Fig. 4a, in the presence of IL-2 (10 U/ml) and IL-15 (50 ng/ml) with or without TGF-β (50 ng/ml). Dot plots depict mean percentage ±SEM of CD117+ cells, CD127+ cells (both *N* = 5) and CD18+ cells (*N* = 2). Each dot represents one donor. **b** Flow cytometry analysis of intracellular expression of cytotoxic molecules granulysin, granzyme B and perforin in population B after culturing for 7 days on OP9neo, which were excluded from the analysis by gating for live CD45+ cells, in the presence of IL-2 (10 U/ml) and IL-15 (50 ng/ml) with or without TGF-β (50 ng/ml). Dot plots depict the mean relative MFI ± SEM of Granulysin (*N* = 6), granzyme B (*N* = 5) and perforin (*N* = 3) upon addition of TGF-β compared to the condition without TGF-β. Each dot represents one donor. **c** Concentration of granulysin in the supernatant of cells from population B cultured on OP9neo for 7 days in the presence of IL-2 (10U/ml) and IL-15 (50 ng/ml) with or without TGF-β (50 ng/ml) measured by ELISA. Dot plot depicts the mean concentration ±SEM, each dot represents one donor (*N* = 3). All Data were tested for normal distribution using the Shapiro-Wilk test and if normally distributed tested with a paired t-test. If not normally distributed (perforin) data were tested with a Wilcoxon matched-paired rank test. All p values are two-sided.

It has been reported that TGF-β can diminish the cytotoxic capacity of NK cells^30,31^, which acts synergistically with IL-15^32^, and it was suggested that this cytokine converted NK cells into ILC1-like cells. Culturing CD16− or CD16+ NK cells in the presence of IL-15 and TGF-β did not induce CD127 or CD117 expression (Fig. S7a). Moreover, granulysin expression was not significantly altered whereas granzyme B and perforin levels were downregulated (Fig. S7b). In contrast, the addition of TGF-β to cultures of CD117− population B cells induced upregulation of CD117 and downregulation of CD18 but did not rescue IL-15 induced CD127 downregulation (Fig. 6a). Furthermore, the addition of TGF-β to the culture downregulated intracellular granulysin, granzyme B and perforin (Fig. 6b) and secretion of granulysin was abrogated by TGF-β (Fig. 6c). Thus IL-15 drives the expression of cytotoxic molecules in CD127+CD94+CD117− granulysin producing ILCs and these effects can be prevented and reverted in the presence of TGF-β.

## Discussion

Analysis of human intestinal NK/ILC pool by single-cell RNA sequencing ILCs and NK cells, sorted by flow cytometry, reveals the existence of conventional NK cells, helper ILC clusters, a cluster resembling LTi cells, and two additional ILC clusters that are characterized by co-expression of CD127 and CD94. These CD127+CD94+ cells were absent in the fetal intestine but expanded in inflamed resection specimen from Crohn’s Disease (CD) patients when compared to normal controls and non-inflamed CD samples This contrasted with NK cells, which did not increase significantly in inflamed CD samples compared to non-inflamed CD samples, indicating that the degree of inflammation in CD patients only affects the pool of CD127+CD94+ cells.

CD127+CD94+ cells which were located in the lamina propria and were therefore distinct from the intraepithelial ILC1 described previously^3^ could be divided into two distinct populations; CD117+CD18dimCD161high population A and CD117-CD18highCD161dim population B. Whereas on protein level CD117 expression correlated with functional and phenotypical features of the two subpopulations, differential *KIT* mRNA expression was not observed in our scRNAseq analysis, providing an example that mRNA levels and protein expression are not always correlated^33,34^. Population A expressed low amounts of effector molecules whereas population B displayed an effector phenotype reflected by the high expression of granulysin and perforin. CD127+CD94+ cells were absent in fetal intestinal tissues whereas mature NK cells were highly abundant, confirming previous observations by us and others^35^. Moreover, whereas mature functional NK cells and ILC3s are present in fetal intestine, co-expression of CD127 and CD94 was previously not found in pediatric small intestine tissues up to 9 months of age^36^. Since ILC3s and NK cells are already present much earlier in life than CD127+CD94+ cells it is unlikely that these latter cells are obligatory precursors of canonical NK cells. This raises the question to what lineage the CD127+CD94+ cells belong. The high expression of CD127, CD161, and CD200R1 in combination with the lack of CD117 in population B are suggestive for an ILC1 identity. Both CD127+CD94+ populations expressed higher *TCF7* and lower *PRDM1* (encoding BLIMP-1) than NK cells. BLIMP-1 is a direct suppressor of TCF7^37^, and TCF7 is implicated to enforce commitment to the innate lymphoid cell lineage^38,39^. TCF7 plays a pivotal role in inhibiting cytotoxic molecules in NK cells and to allow for normal expansion and function^40^. The higher expression of TCF7 observed in the CD127+CD94+ populations may explain the lower expression of granzymes and could support an ILC lineage commitment, but the expression of *KLRF1* (encoding NKp80), Eomes and cytotoxic molecules would be more consistent with an NK cell identity. It might be possible that the intestinal CD127+CD94+ cells are related to peripheral blood CD56bright CD16- NK cells that weakly express CD127 and that may be precursors of CD56dim CD16+ NK cells^41^. However, we did not observe differentiation of CD127+CD94+ towards CD16+ NK cells. Our data are consistent with the notion that population B arise from population A and we speculate that population A arise from helper ILCs. Recently we identified RORγt+ ILCs expressing Eomes and cytotoxic molecules in tonsils^10^. Those latter data and those presented here strongly suggest that ILC subsets can acquire a cytotoxic machinery. Those cytotoxic ILCs may be comparable to cytotoxic CD4+ T cells^42–45^.

The unique property of CD127+CD94+ population B to express and secrete high protein levels of granulysin indicated that these cells are activated. Although granulysin was secreted, the expression of intracellular granulysin remained very high, suggesting a constant production and refill of granulysin within these cells. Granulysin was shown to induce bacterial lysis, either alone in high concentrations, or synergistically with granzyme B or perforin^12–16^. Defects in the epithelial barrier during active inflammation in Crohn’s disease patients can result in infiltration of bacteria into the lamina propria. Therefore, expansion of CD127+CD94+ granulysin-expressing cells may be beneficial by combatting these infiltrating bacteria. On the other hand, granulysin may also act as an immune alarmin that induces the recruitment and maturation of dendritic cells^46^, including the activation of pathways needed to induce Th1 development^47^. Migration of both CD4 and CD8 T cells, NK cells and monocytes in response to granulysin has also been reported^48^. Furthermore, our data revealed co-expression of *SELL* (CD62L) and *CCR7* in granulysin high cells indicating that these cells are capable of traveling to the lymph node via high endothelial venules^49^, where they may efficiently attract other immune cells to the site of inflammation via secretion of granulysin. Besides granulysin, also granzyme B, A and K are implicated in boosting inflammation by enhancing the release of pro-inflammatory TNF-α by myeloid cells in synergy with LPS^50^. Additionally, granzyme B cleaves pro-IL-18 and the IL-1α precursor^51^, cytokines that are elevated in Crohn’s disease patients^24,25^. These properties could eventually exacerbate inflammation and thus the observed elevated frequencies of CD127+CD94+ cells could be detrimental to the patient. Given that IL-15 is highly expressed in patients with inflammatory bowel disease^52^, secretion of granulysin by chronically activated CD127+CD94+ cells can contribute to inflammation. Exposing granulysin high cells to anti-inflammatory TGF-β diminished the secretion of granulysin, even in presence of IL-15, and dampened the levels of intracellular granzyme B and perforin and may thus intervene in inflammation. TGF-β has been described to diminish cytotoxic capacities in NK cells^30,31^, in synergy with IL-15^32^ and these NK cells displayed an ILC1-like phenotype. However, treatment of NK cells with IL-15 and TGF-β did not induce CD127 expression or granulysin downregulation. It remains to be elucidated whether these cells can become non-cytotoxic ILC1s as described previously^18,26^. TGF-β did also not significantly upregulate granulysin or CD117 in NK cells, indicating that these TGF-β-treated NK cells are distinct from granulysin high CD117- population B cells or CD117+ population A cells.

All together, we identified an ILC1-like cell type that stably expresses high levels of the cytotoxic molecules granulysin and perforin, but not granzyme B. The observed expansion of these ILC1s in Crohn’s disease patients may be detrimental to the patients by enhancing inflammation but could also be beneficial by the killing of bacteria via granulysin secretion. Cytotoxic ILC1-like cells have been described in the mouse^53^. These cells which were shown to be involved in immune surveillance against cancer^53^ may be similar to CD94+CD127+ cells described here. This raises the possibility that the CD127+CD94+ ILC1 identified here are involved in anti-cancer immune responses.

## Methods

### Tissue collection

All tissues were collected after subjects provided informed consent, with approval of tissue specific protocols by the Medical Ethical committee of the Amsterdam UMC. Intestinal ileum or colon was obtained after surgical resection with the exclusion of subjects that had undergone chemo- or radiotherapy prior to surgery. Inflammation status was macroscopically determined by the pathologist. Human fetal intestine was obtained from abortions at the Stichting Bloemenhoven clinic in Heemstede, the Netherlands, upon informed consent and approval of the Medical Ethical Committee of Amsterdam UMC. Gestational age, determined by ultrasonic measurement of the skull or femur diameter, ranged from 14 to 19 weeks.

### Isolation of ILCs from intestinal tissue

Intestinal ileum or colon was processed to obtain cell suspensions^54^. Intestinal lamina propria was washed extensively with PBS and subsequently incubated for 30 min with PBS+5 mM EDTA at 4 °C to separate epithelial cells from the lamina propria. Lamina propria was then cut into small pieces and digested for 30 min at 37 °C with RPMI (GIBCO)+Liberase TM (125 μg/ml) + DNAse I (0.1 mg/mL). Fetal intestinal samples were cut longitudinally, cleaned and cut into small pieces, followed by enzymatic digestion using IMDM+Liberase TM (125 µg/ml) + DNase I (0,1 mg/ml) for 1 h. Obtained cell suspensions were filtered through a 70 µm cell strainer, treated with red cell lysis buffer and stained for 30 min at 4 °C with fluorochrome-conjugated antibodies. All antibodies used for this study are listed in table S2. The cells were sorted on a FACSAria IIU (BD Biosciences) either in bulk, or as single cells in a 384 wells plate for single-cell RNA sequencing.

### Flow cytometry analysis

For flow cytometry analysis, cells were stained for surface antigens and fixable viability dye for 30 min at 4 °C in PBS. For intracellular stainings cells were washed and fixed using 4% paraformaldehyde for 10 min, followed by permeabilization using the Foxp3/Transcription factor staining buffer kit (ThermoFisher Scientific). Samples were subsequently stained with intracellular cytokine/transcription factor antibodies for 30 min at room temperature in permeabilization buffer. Samples were acquired on the LSRFortessa or FACSAria IIU (BD Biosciences) and analyzed using FlowJo software (FlowJo LLC, Ashland, OR)

### ILC/NK cell co-culture with OP9 cells

Naïve OP9 murine stromal cells were kindly provided by Dr. T. Nakano (Osaka University, Osaka, Japan). For co-cultures with ILCs or NK cells, 3000 OP9 cells were plated at least 5 h prior to co-culture per well in a 96-well round bottom plate in Yssel’s medium (IMDM+4% (v/v) Yssel’s supplement (made in house, Amsterdam UMC) +1% (v/v) human AB serum (Invitrogen)). For bulk cultures (500-2000 ILCs per condition), ILCs or NK cells were cultured with OP9 cells for 7 days with IL-2 (10 U/ml) with combinations of, IL-12, IL-23, IL-1β, IL-15 and TGF-β (all 50 ng/ml) and cytokines were supplemented at day 3 and 6. Only for IL-22 and IFN-y expression, cells were stimulated with PMA (10 ng/ml; Sigma) plus Ionomycin (500 nM; Merck) in presence of Golgi Plug (555029;BD) for 3 h prior to FACS analysis.

### Granulysin secretion analysis

Supernatants were collected after culturing and analyzed for the presence of granulysin protein by Enzyme-Linked Immunosorbent Assay (ELISA) according to the manufacturer’s protocol (R&D systems, catalog number DY3138).

### Quantitative real-time PCR

Sorted cell populations were lysed and RNA was isolated using the NucleoSpin RNA XS kit (Macherey-Nagel) according to the manufacturer’s protocol. Complementary DNA was synthesized using the High-Capacity Archive kit (Applied Biosystems). PCR was performed with a LightCycler 480 Instrument II (Roche) with SYBR Green I master mix (Roche). Primers sets that were used are listed in Table S3 Analysis of expression was performed with Bio-Rad CFX Manager 3.1 software and normalized to the expression of β-actin.

### Single-cell RNA sequencing

#### CEL-Seq2-based single-cell RNA sequencing

For single-cell RNA sequencing, we isolated lamina propria mononuclear cells from inflamed ileum that was surgically removed from an individual with Crohn’s disease. Isolation was performed directly after surgery. Detailed patient information can be found in supplementary table 1. 2304 cells were directly sorted in 384-well plates, frozen and sent to Single Cell Discoveries for further processing of their pipeline based on CEL-Seq2^55,56^. Libraries were run on a HiSeq4000 for Illumina sequencing. Post-processing and quality control were performed by Single Cell Discoveries. Reads were aligned to GRCh38 reference assembly using STARsolo^57^. Primary assessment reported 2,441 median unique molecular identifiers (UMIs, transcripts) per cell and 1093 median genes per cell sequenced to 91.7% sequencing saturation with 35,019 mean reads per cell.

#### Data analysis

All transcriptome data analysis was performed in RStudio running R. For the scRNAseq the Seurat package was used in combination with the tidyverse package. Adjusted P values were calculated using the Benjamini–Hochberg method. Data were visualized using Seurat v4, glimma and tidyverse R packages^58–65^.

### Reporting summary

Further information on research design is available in the Nature Research Reporting Summary linked to this article.

## Supplementary information

Supplementary Information

Peer Review File

Reporting summary

## Data Availability

Single-cell RNA sequencing data that support the findings of this study will be deposited in GEO with the accession code GSE173642 (http://www.ncbi.nlm.nih.gov/geo/GSE173642)”
