# Supplementary material for: CD127+ CD94+ innate lymphoid cells expressing granulysin and perforin are expanded in patients with Crohn’s disease
Source: Nat Commun. 2021 Oct 6;12:5841. doi: 10.1038/s41467-021-26187-x (PMC8494908; doi:10.1038/s41467-021-26187-x)
Supplement: Supplementary file 1 — Supplementary Information [file 41467_2021_26187_MOESM1_ESM.pdf]

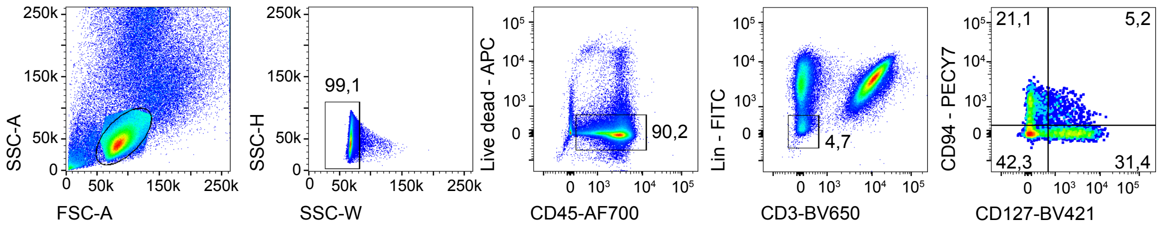

Supplementary figure 1. Gating strategy used for sorting of human intestinal ILCs and NK cells

ILCs and NK cells were gated on viable CD45+Lineage-(CD1a, CD3, CD4, CD5, CD14, CD19, CD34, CD123, CRTH2, BDCA2, TCRαβ, TCRγδ and FcER1α) and subsequently on CD127+ for total ILCs and on CD127-CD94+ for NK cells.

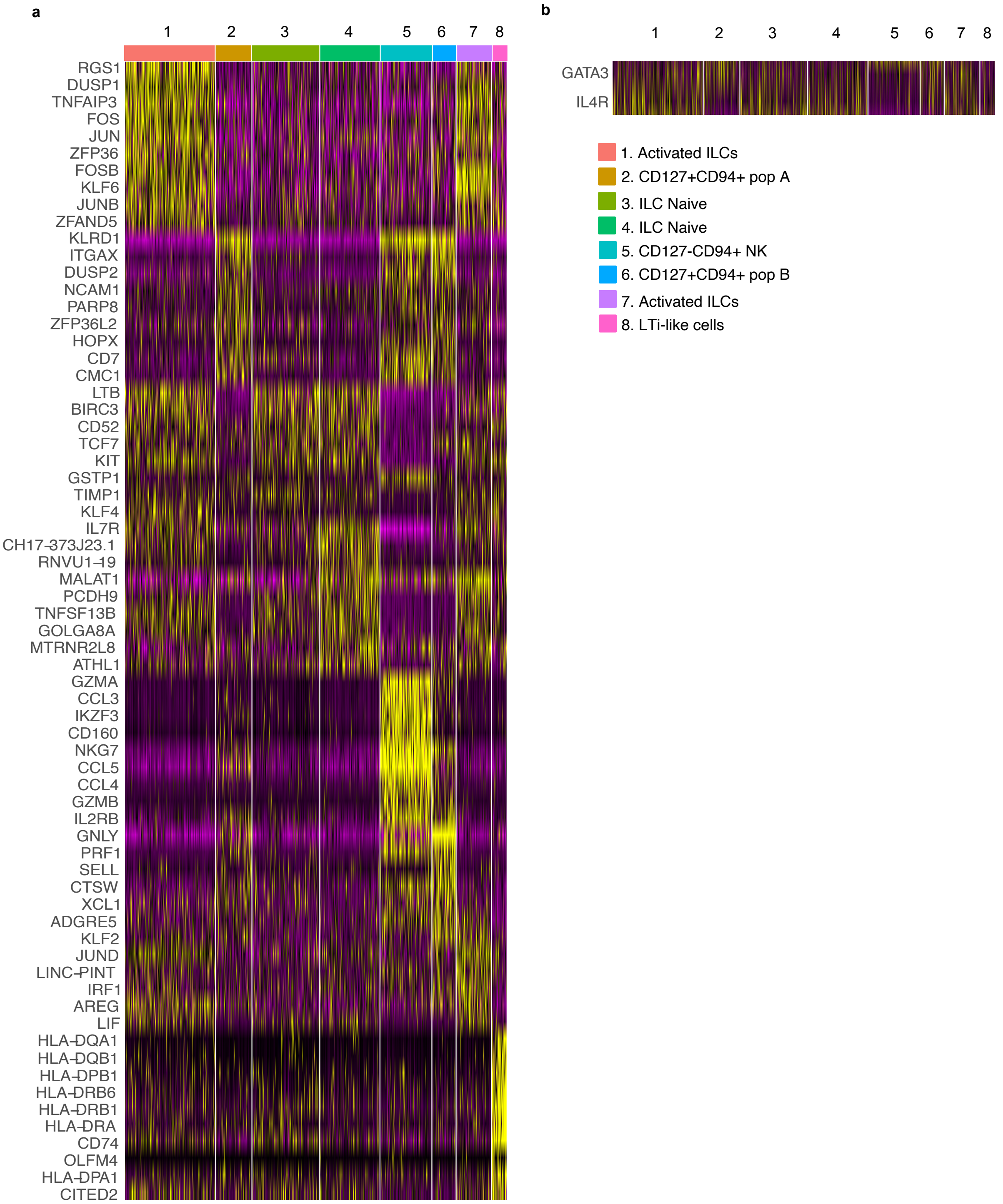

Supplementary figure 2. Most differentially expressed genes in ILC/NK clusters found by scRNAseq

a. Heatmap depicting 10 most differentially expressed genes in clusters indicated in Fig. 1B.

b. Heatmap of the expression of indicated genes in clusters indicated in Fig. 1B.

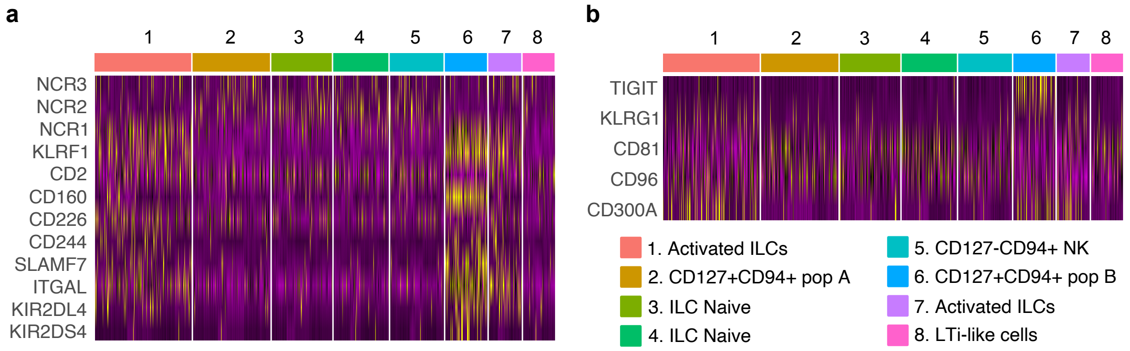

Supplementary figure 3. Expression of activating and inhibitory receptors NK/ILC clusters.

a-b. Heatmap of the expression of indicated genes in clusters indicated in Fig. 1.

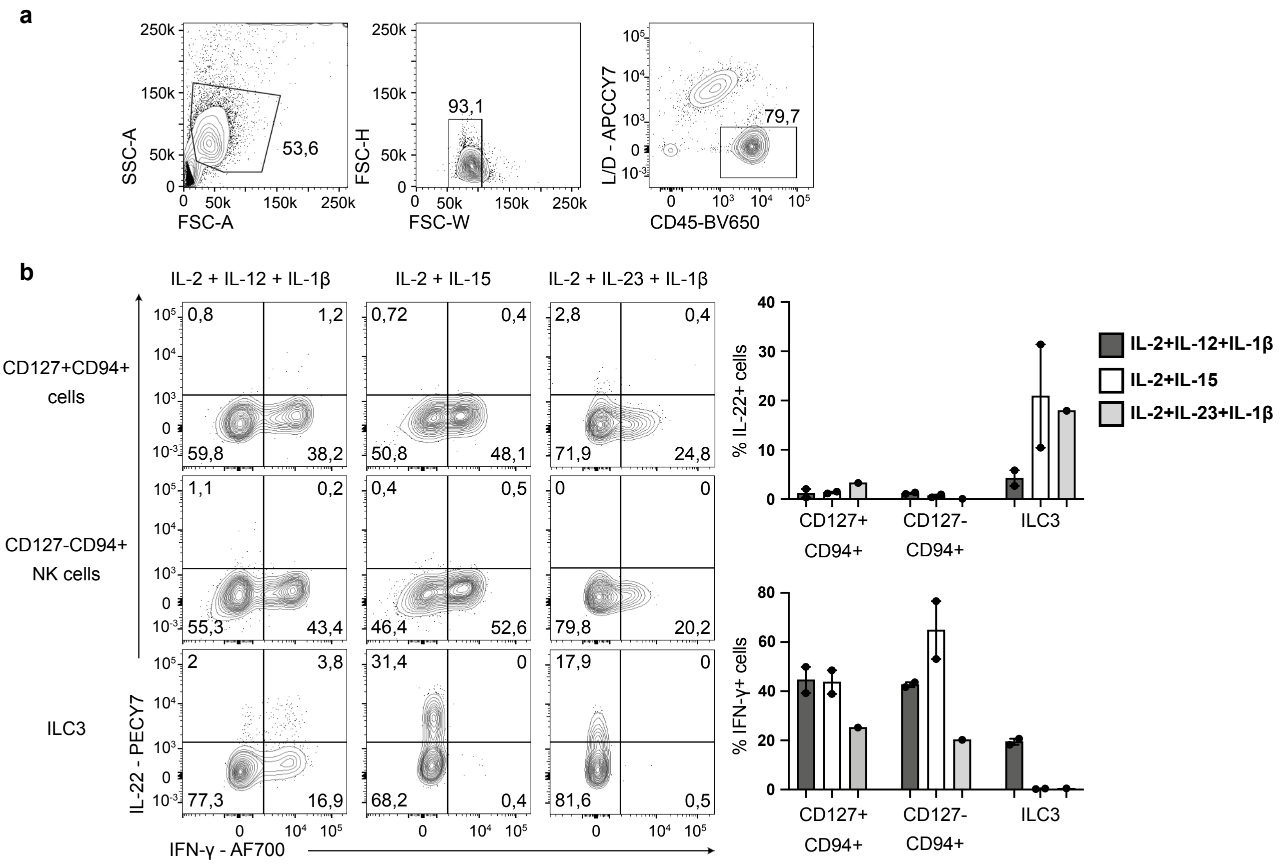

Supplementary figure 4. CD127+CD94+ cells express IFN-γ but not IL-22

a. Gating strategy for the analysis of ILCs / NK cells upon culturing on OP9.

b. Flow cytometry analysis of intracellular expression of IL-22 and IFN-γ in CD127+CD94+ cells, NK cells and ILC3s after culturing for 7 days on OP9, which were excluded from the analysis by gating for live CD45+ cells, in the presence of IL-2 (10 U/ml) plus combinations of IL-12, IL-23, IL-*1*β *or* IL-15 (all 50 ng/ml). Bar graphs show the percentage ±SEM of IL-22 and IFN-γ expressing cells. Each dot represents one donor (N=2 for stimulation with IL-2+IL-15 and IL-2+IL-12+IL-1β, and N=1 for stimulation with IL-2+IL-23+IL-1β).

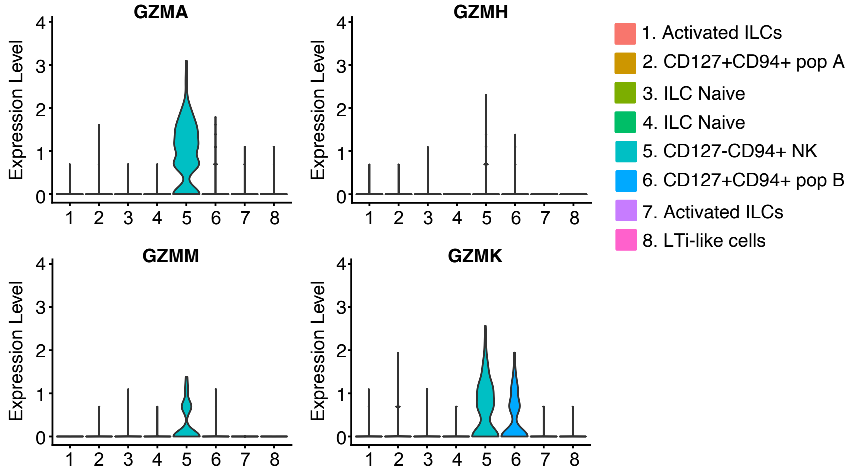

Supplementary figure 5. NK cells express more granzymes than CD127+CD94+ populations

Violin plots showing the mRNA expression of GZMA, GZMA and GZMM in indicated populations.

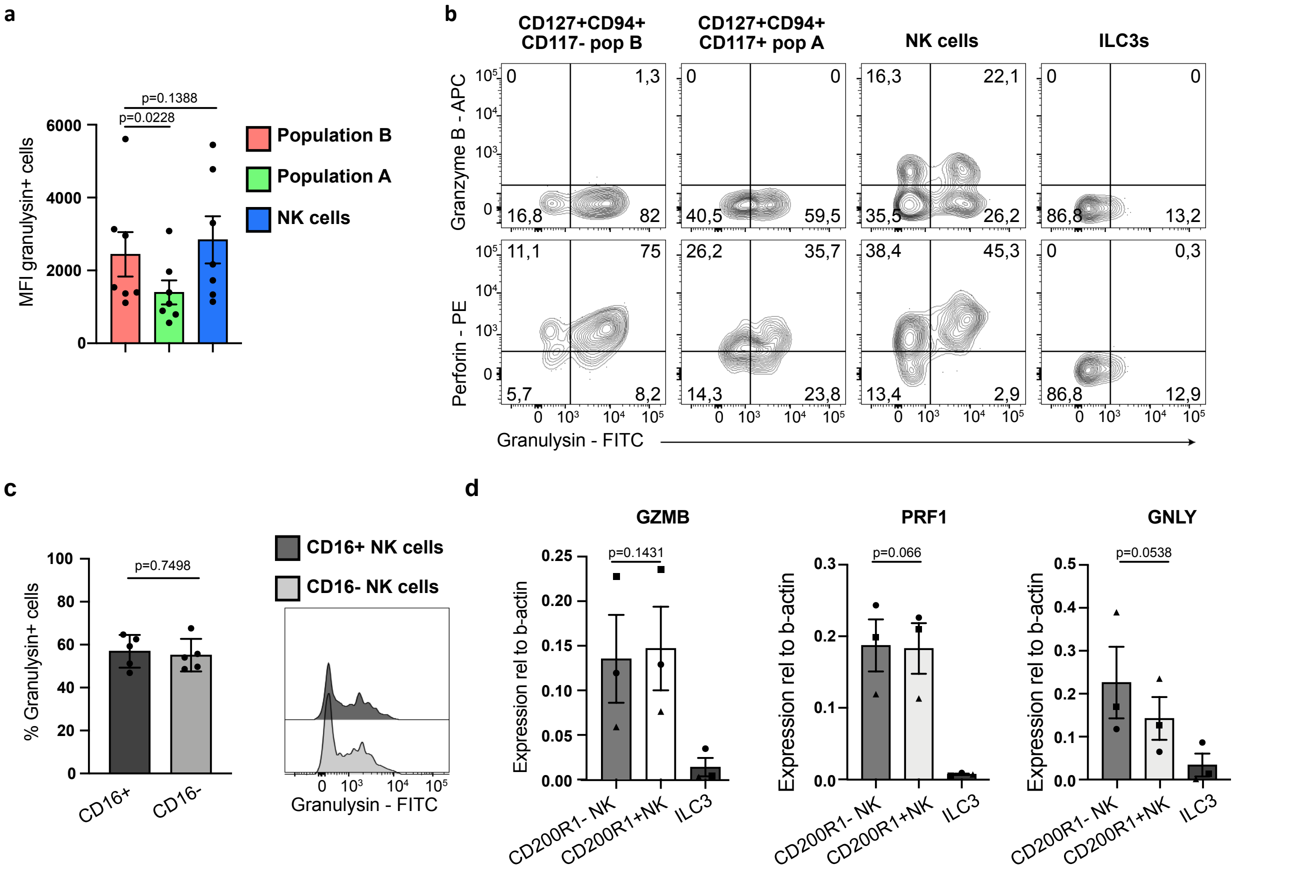

Supplementary figure 6. Granulysin MFI is similar between NK cells and population B and the frequency of population B co-expressing granulysin and perforin is higher than in NK cells

a. Bar graph depicts the MFI ±SEM of intracellular granulysin protein expression (N=7) within the granulysin+ cells of indicated subsets directly measured after isolation.

b. Representative flow cytometry analysis of intracellular protein expression of granulysin (N=7), granzyme B (N=4) and perforin (N=3) in indicated populations.

c. Flow cytometry analysis of intracellular granulysin protein expression in CD16- and CD16+ NK cells. Dot plot show the mean percentage ±SEM positive cells, each dot represents one donor (N=5) and data was tested for normal distribution using the Shapiro-Wilk test and tested with a paired t-test. ns = not significant

d. mRNA expression of PRF1, GZMB and GNLY in CD200R1- NK cells, CD200R+ NK cells and CD117+ ILC3s measured by RT-qPCR. Dot plots show the mean expression ±SEM relative to β-actin, each dot represents one donor (N=3).

All data was tested for normal distribution using the Shapiro-Wilk test and tested with a paired t-test. All p values are two-sided.

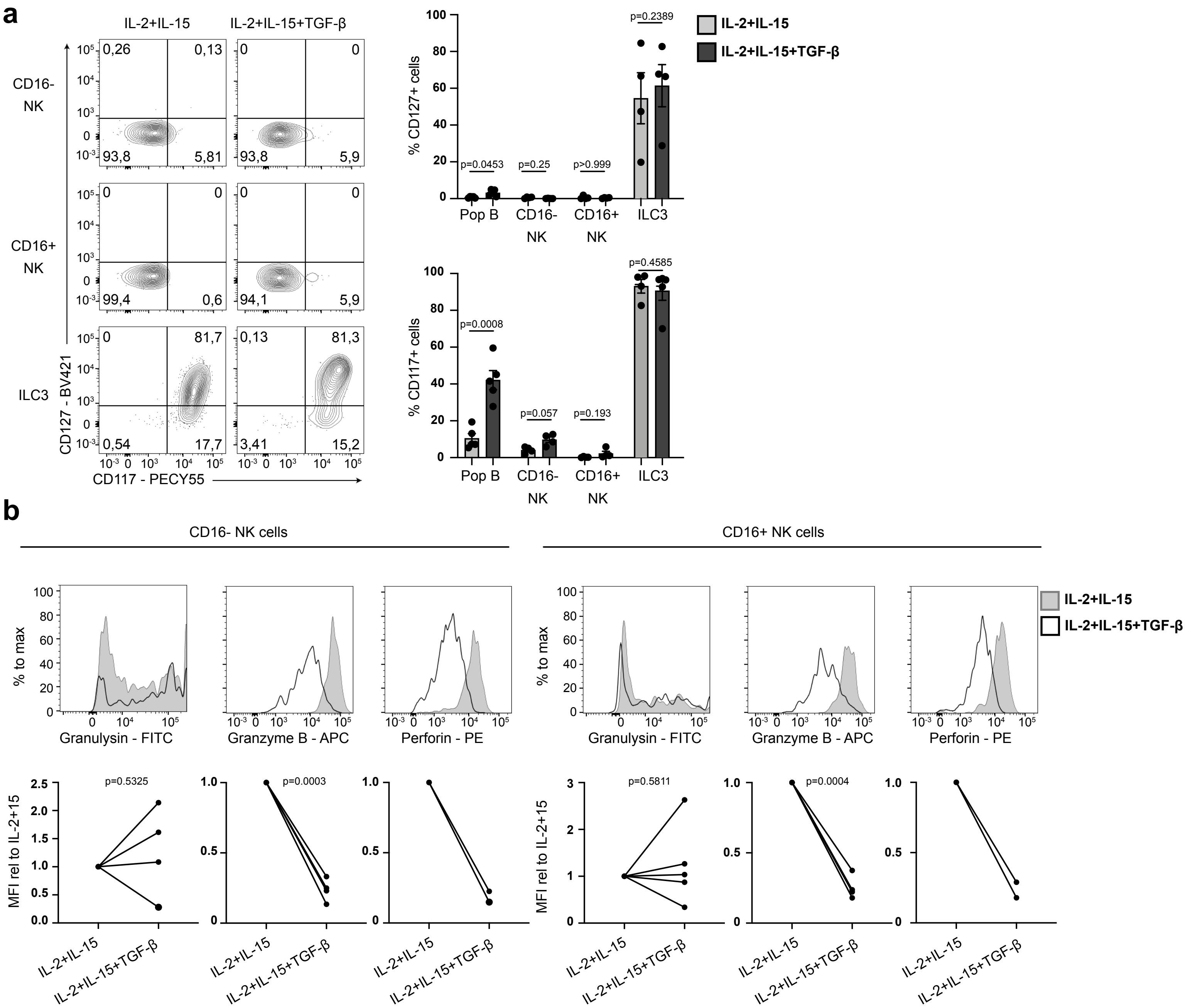

Supplementary figure 7. NK cells do not upregulate helper ILC markers in culture

a. Flow cytometry analysis of cultured cells from CD16- NK (CD127-CD94+), CD16+ NK and ILC3s (CD127+CD94-CD117+) for the expression of CD117 and CD127. Cells were cultured for 7 days on OP9, which were excluded from the analysis by gating for live CD45+ cells, in the presence of IL-2 (10U/ml) and IL-15 (50 ng/ml), with or without TGF-*β* (50 ng/ml). Dot plots depicts the mean percentage ±SEM of CD117+ and CD127+ cells after culturing. Each dot represents one donor (N=4)

b. Flow cytometry analysis of intracellular expression of cytotoxic molecules granulysin, granzyme B and perforin in CD16- NK cells and CD16+ NK cells after culturing for 7 days on OP9, which were excluded from the analysis by gating for live CD45+ cells, in the presence of IL-2 (10 U/ml) and IL-15 (50 ng/ml) with or without TGF-*β* (50 ng/ml). Bar graphs show the mean relative MFI ±SEM of Granulysin (N=5), granzyme B (N=4) and perforin (N=2) upon addition of TGF-*β* compared to the condition without TGF-*β*. Each dot represents one donor.

Data was tested for normal distribution using the Shapiro-Wilk test and tested with a paired t-test. * P<0,05, *** P<0,001, ns = not significant. All p values are two-sided.

Supplementary table 1. Patient characteristics

patient ID	Age	Gender	Smoking	Diagnosis	Surgery	Montreal Classification - Disease Location	Montreal Classification - Disease behavior	Montreal - perianal disease modifier	Reason for surgery	Deviation present	IBD - Medications	Site of sample collection	Part
**Normal group**													
normal 1	74	M	no	Radiation enteritis	Colectomy	NA	NA	NA	Complaints incontinence	No	No		colon
normal 2	67	M	no	Fasciitis necroticans	Abdominal extirpation	NA	NA	NA	Fasciitis necroticans	No	No		colon
normal 3	46	F	yes	slow-transit colon	Subtotal colectomy	NA	NA	NA	slow-transit colon	No	No		ileum
**UC**													
UC 1	53	M	no	Ulcerative Colitis	Removal stoma	NA	NA	NA	Wish to remove ileostomy	Yes	No	Non-inflamed	ileum
UC 2	78	F	yes	Ulcerative Colitis	Hemi-colectomy	NA	NA	NA	Dysplasia	No	No	Non-inflamed	ileum
UC 3	32	F	yes	Ulcerative Colitis	Pouch	NA	NA	NA	Wish for IPAA	Yes	No	Non-inflamed	ileum
**CD non infl**													
CD non infl 1	26	M	no	Crohn's disease	Ileocecal resection	1	2	0	Stenosis and perforation terminal ileum	No	No	Non-inflamed	Ileum
CD non infl 2	30	F	no	Crohn's disease	Colectomy	3	3	1	Surgery due to persistend complaints Crohn's disease activity and stenosis sigmoid	Yes	No	Non-inflamed	Colon
CD non infl 3	74	F	no	Crohn's disease	Ileocecal resection	1	2	0	Stenosis neo-terminal ileum	No	No	Non-inflamed	ileum
CD non infl 4	37	F	yes	Crohn's disease	Colostomy	3		1	Terminal colostomy	No	Thiopurines	Non-inflamed	Colon
**CD inflamed**													
CD infl 1	65	F	no	Crohn's disease	Ileocecal resection	1	2	0	Stenosis neo-terminal ileum	Yes	Steroid local	inflamed	ileum
CD infl 2	19	M	yes	Crohn's disease	Ileocecal resection	3	2	1	active disease	No	Anti-TNF, thiopurines	inflamed	ileum
CD infl 3	31	F	no	Crohn's disease	Proctectomy	3	2	1	active disease, therapy-resistant	Yes	Ustekinumab	inflamed	Rectum
CD infl 4	33	M	no	Crohn's disease	Hemi-colectomy	3	2+3		Enterocutanous fistula in old appendectomyscar by Crohn's Disease	Yes	None	inflamed	colon
CD infl 5	36	F	yes	Crohn's disease	Colectomy	2	3	1	active disease, therapy refractory	Yes	Anti-TNF, steroids	inflamed	colon
CD infl 6	63	M	no	Crohn's disease	Proctocolectomy	3	1	0	active disease	Yes	none	inflamed	ileum
CD infl 7	49	M	no	Crohn's disease	Ileocecal resection	1	2	0	Stenosis and ileitis terminalis with fistulinzg disease to sigmoid and proximal small intestine	No	none	inflamed	ileum
CD infl 8	29	M	no	Crohn's disease	Ileum resection	4	2		stenosis	No	anti-TNF	inflamed	ileum
CD infl 9	25	M	no	Crohn's disease	Stricturoplasty	3	2	0	stenosis	No	anti-TNF	inflamed	ileum
CD infl 10	20	M	no	Crohn's disease	Ileocecal resection	3	3	0	Perforated ileitis terminalis	No	thiopurines,	inflamed	ileum
CD infl 11	28	F	no	Crohn's disease	Ileocecal resection	3	3	1	active disease with multiple abcesses	No	anti-TNF	inflamed	ileum
CD infl 12	30	F	yes	Crohn's disease	Ileocecal resection	3	3	0	Perforated ileitis terminalis	No	anti-TNF	inflamed	ileum
CD infl 13 Same pt as CD non infl 3	74	F	no	Crohn's disease	Ileocecal resection	1	2	0	Stenosis neo-terminal ileum	No	none	inflamed	ileum
CD infl 14	64	F	yes	Crohn's disease	Ileocecal resection		2	0	Clinical stenosis	No	Steroids systemic	inflamed	colon
CD infl 15	25	F	no	Crohn's disease	Ileocecal resection	1	3	0	Ileitis terminalis with abcessess	No	none	inflamed	ileum
CD infl 16	33	F	no	Crohn's disease	Ileum resection	2	2		stenosis	No	Vedolizumab, systemic	inflamed	colon
CD infl 17	56	F	no	Crohn's disease	Ileum resection	3	2	1	stenosis	No	Anti-TNF, thiopurines	inflamed	ileum
CD infl 18	52	F	no	Crohn's disease	Ileocecal resection	1	2		Clinical stenosis	No	none	inflamed	ileum

Used for single cell RNA sequencing

CD infl 7 (see above)	Fresh

Supplementary table 2. All antibodies used for the study

Antigen (all human)	Clone	Fluorochrome	Company	#Cat	#Lot	Dilution
**CD1a**	HI149	FITC	Biolegend	300104	B214337	1:50
**CD3**	OKT3	FITC	Biolegend	317306	B278881	1:50
**CD3**	UCHT1	AF700	Biolegend	300424	B252009	1:50
**CD4**	RPA-T4	FITC	Biolegend	300506	B225958	1:50
**CD14**	HCD14	FITC	Biolegend	325604	B285084	1:50
**CD19**	HIB19	FITC	Biolegend	302206	B274550	1:50
**CD34**	581	FITC	Biolegend	343504	B252495	1:50
**CD123**	6H6	FITC	Biolegend	306014	B273854	1:50
**CRTH2**	BM16	FITC	Biolegend	350108	B176843	1:20
**BDCA2**	201A	FITC	Biolegend	354208	B212274	1:50
**TCRαβ**	IP26	FITC	Biolegend	306706	B273449	1:50
**TCRγδ**	B1	FITC	Biolegend	331208	B226717	1:50
**FcER1α**	AER-37 (CRA-1)	FITC	Biolegend	334608	B266717	1:50
**CD45**	2D1	APCCy7	Biolegend	368518	B248068	1:100
**CD45**	HI30	BV605	Sony	2120210	109942	1:100
**CD127**	A01905	PECy7	Biolegend	351320	B251081	1:20
**CD161**	HP-3G10	BV510	Biolegend	339922	B231692	1:50
**CD94**	HP-3D9	PE	BD biosciences	555889	7174991	1:50
**CD94**	HP-3D9	BV510	BD biosciences	743949	0232028	1:50
**CD94**	DX22	FITC	Biolegend	305905	B199409	1:50
**CD56**	HCD56	BV605	Biolegend	362538	B280386	1:200
**CD16**	3G8	APCCy7	Biolegend	302060	B322021	1:100
**CD16**	3G8	BV785	Biolegend	302046	B259007	1:100
**CD117**	104D2D1	PECy5.5	Beckman coulter	B96754	200022	1:100
**NKp44**	44.189	BV421	ebioscience	48-3369-42	4318961	1:100
**NKp80**	5D12	PE	Biolegend	346706	B202427	1:20
**NKp80**	5D12	APC	Biolegend	346708	B273989	1:20
**CD200R1**	OX-108	BV421	BD biosciences	566344	0044846	1:50
**CD200R1**	OX-108	PETx	Biolegend	329310	B285843	1:50
**NKp46**	9E2	BV421	Biolegend	331914	B241579	1:100
**IL-1R1**	Polyclonal Goat IgG	PE	R&D systems	FAB269p	AAFG0309071	1:100
**EOMES**	WD1928	PETx	ebioscience	12-5899-73	E019967	1:50
**RORγt**	Q21-559	APC	BD biosciences	563620	9101972	1:50

**Granzyme B**	GB11	AF647	Biolegend	515405	B197353	1:30
**Granulysin**	RB1	FITC	BD biosciences	558254	5582545	1:15
**Perforin**	B-D48	PE	Biolegend	353304	B292339	1:30
**IFN-γ**	B27	AF700	Biolegend	506516	B290145	1:50
**IL-22**	22URTI	PECY7	Invitrogen	25-7229-42	2008218	1:50

Supplementary table 3. Primer sets used for RT-qPCR

**Gene**	**Forward Primer**	**Reverse primer**
B-actin	CACCATTGGCAATGAGCGGTTC	AGGTCTTTGCGGATGTCCACGT
GRZB	CGACAGTACCATTGAGTTGTGCG	TTCGTCCATAGGAGACAATGCCC
PRF1	ACTCACAGGCAGCCAACTTTGC	CTCTTGAAGTCAGGGTGCAGCG
GNLY	GTACTACGACCTGGCAAGAGCC	TCAGACAGGTCCTGTAGTCACG
