# Supplementary material for: CD127+ CD94+ innate lymphoid cells expressing granulysin and perforin are expanded in patients with Crohn’s disease
Source: Nat Commun. 2021 Oct 6;12:5841. doi: 10.1038/s41467-021-26187-x (PMC8494908; doi:10.1038/s41467-021-26187-x)
Supplement: Supplementary file 3 — Reporting summary [file 41467_2021_26187_MOESM3_ESM.pdf]

nature research

Corresponding author(s): Prof. H. Spits

Last updated by author(s): 31-08-2021

Reporting Summary

Nature Research wishes to improve the reproducibility of the work that we publish. This form provides structure for consistency and transparency in reporting. For further information on Nature Research policies, see our Editorial Policies and the Editorial Policy Checklist.

Statistics

For all statistical analyses, confirm that the following items are present in the figure legend, table legend, main text, or Methods section.

n/a	Confirmed	
☐	☒	The exact sample size (*n*) for each experimental group/condition, given as a discrete number and unit of measurement
☐	☒	A statement on whether measurements were taken from distinct samples or whether the same sample was measured repeatedly
☐	☒	The statistical test(s) used AND whether they are one- or two-sided<br>*Only common tests should be described solely by name; describe more complex techniques in the Methods section.*
☐	☒	A description of all covariates tested
☐	☒	A description of any assumptions or corrections, such as tests of normality and adjustment for multiple comparisons
☐	☒	A full description of the statistical parameters including central tendency (e.g. means) or other basic estimates (e.g. regression coefficient) AND variation (e.g. standard deviation) or associated estimates of uncertainty (e.g. confidence intervals)
☐	☒	For null hypothesis testing, the test statistic (e.g. *F*, *t*, *r*) with confidence intervals, effect sizes, degrees of freedom and *P* value noted<br>*Give P values as exact values whenever suitable.*
☒	☐	For Bayesian analysis, information on the choice of priors and Markov chain Monte Carlo settings
☒	☐	For hierarchical and complex designs, identification of the appropriate level for tests and full reporting of outcomes
☒	☐	Estimates of effect sizes (e.g. Cohen's *d*, Pearson's *r*), indicating how they were calculated

Our web collection on statistics for biologists contains articles on many of the points above.

Software and code

Policy information about availability of computer code

Data collection	BD FACS diva software<br>CFX Maestro Software
Data analysis	R version 4.0.5<br>RStudio version 1.4.1106<br>Seurat version 4<br>Flowjo 10.7.1<br>Graphpad prism 8<br>CFX Maestro Software

For manuscripts utilizing custom algorithms or software that are central to the research but not yet described in published literature, software must be made available to editors and reviewers. We strongly encourage code deposition in a community repository (e.g. GitHub). See the Nature Research guidelines for submitting code & software for further information.

Data

Policy information about availability of data

All manuscripts must include a data availability statement. This statement should provide the following information, where applicable:

- Accession codes, unique identifiers, or web links for publicly available datasets
- A list of figures that have associated raw data
- A description of any restrictions on data availability

Single cell RNA sequencing data that support the findings of this study are deposited in GEO with the accession code GSE173642 (http://www.ncbi.nlm.nih.gov/geo/GSE173642)"]

Field-specific reporting

Please select the one below that is the best fit for your research. If you are not sure, read the appropriate sections before making your selection.

[x] Life sciences   [ ] Behavioural & social sciences   [ ] Ecological, evolutionary & environmental sciences

For a reference copy of the document with all sections, see nature.com/documents/nr-reporting-summary-flat.pdf

Life sciences study design

All studies must disclose on these points even when the disclosure is negative.

Sample size	Sample size was determined on availability of intestinal tissue
Data exclusions	no data was excluded
Replication	All experiment replicates shown in the manuscript are performed in independent experiments (one donor at the time). All performed experiments are shown in the manuscript
Randomization	Experimental groups were determined first by disease status, which was determined by the clinic. Inflammation status was determined by the pathologist based on macroscopic inflammation of the intestinal tissue.
Blinding	investigators were not blinded as the inflammation status of the specimen was communicated to the researcher when tissue was obtained.

Reporting for specific materials, systems and methods

We require information from authors about some types of materials, experimental systems and methods used in many studies. Here, indicate whether each material, system or method listed is relevant to your study. If you are not sure if a list item applies to your research, read the appropriate section before selecting a response.

Materials & experimental systems

n/a	Involved in the study	
[ ]	[x]	Antibodies
[x]	[ ]	Eukaryotic cell lines
[x]	[ ]	Palaeontology and archaeology
[x]	[ ]	Animals and other organisms
[ ]	[x]	Human research participants
[x]	[ ]	Clinical data
[x]	[ ]	Dual use research of concern

Methods

n/a	Involved in the study	
[x]	[ ]	ChIP-seq
[ ]	[x]	Flow cytometry
[x]	[ ]	MRI-based neuroimaging

Antibodies

Antibodies used	All antibodies used for the study are listed in the manuscript, supplemental table 2
Validation	All antibodies used for the study are commercially available and validation data can be found on the manufacterer's website. The manufacturer and the product number of each antibody can be found in the manuscript in supplementary table 2.

Human research participants

Policy information about studies involving human research participants

Population characteristics	Detailed information on the human research participants is listed in the manuscript in supplementary table 1.
Recruitment	Intestinal ileum or colon was obtained from each subject that had given informed consent and when sufficient tissue was left after surgical resection. We only excluded subjects that had undergone chemo- or radiotherapy prior to surgery.
Ethics oversight	All intestinal tissues were collected after subjects provided informed consent, with approval of tissue specific protocols by the Medical Ethical committee of the Amsterdam UMC. Intestinal ileum or colon was obtained after surgical resection with the exclusion of subjects that had undergone chemo- or radiotherapy prior to surgery.<br><br>Human fetal intestine was obtained from abortions at the Stichting Bloemenhoven clinic in Heemstede, the Netherlands, upon informed consent and approval of the Medical Ethical Committee of Amsterdam UMC

Note that full information on the approval of the study protocol must also be provided in the manuscript.

Flow Cytometry

Plots

Confirm that:

- [x] The axis labels state the marker and fluorochrome used (e.g. CD4-FITC).
- [x] The axis scales are clearly visible. Include numbers along axes only for bottom left plot of group (a 'group' is an analysis of identical markers).
- [x] All plots are contour plots with outliers or pseudocolor plots.
- [x] A numerical value for number of cells or percentage (with statistics) is provided.

Methodology

Sample preparation	Intestinal ileum or colon was processed to obtain cell suspensions as described before54. Intestinal lamina propria was washed extensively with PBS and subsequently incubated for 30 minutes with PBS + 5mM EDTA at 4°C to separate epithelial cells from the lamina propria. Lamina propria was then cut into small pieces and digested for 30 min at 37°C with RPMI (GIBCO) + Liberase TM (125 µg/ml) + DNAse I (0.1 mg/mL). Fetal intestinal samples were cut longitudinally, cleaned and cut into small pieces, followed by enzymatic digestion using IMDM + Liberase TM (125 µg/ml) + DNase I (0,1 mg/ml) for 1 hour. Obtained cell suspensions were filtered through a 70 µm cell strainer, treated with red cell lysis buffer and stained for 30 min at 4°C with fluorochrome-conjugated antibodies.
Instrument	FACSAria IIU (BD Biosciences), LSRFortessa (BD Biosciences)
Software	FlowJo software V10.7.1 (FlowJo LLC, Ashland, OR), FACS Diva
Cell population abundance	Due to low cell numbers, purity was checked randomly in some samples. Sorted fractions were pure as no outgrowth of other cell types were found in the expansion cultures.
Gating strategy	Lymphocyes gate was placed in fsc ssc plots. Then selected for single cells by plotting ssc-h vs ssc-w. Live CD45+ cells were selected for further gating.

- [x] Tick this box to confirm that a figure exemplifying the gating strategy is provided in the Supplementary Information.
